# Pasteurellaceae [pas′′-tər-ə-lā′-sē-ī]

**DOI:** 10.3201/eid3010.240735

**Published:** 2024-10

**Authors:** Clyde Partin

**Affiliations:** Emory University School of Medicine, Atlanta, Georgia, USA

**Keywords:** Pasteurellaceae, Louis Pasteur, etymology, bacteria, pasteurization

A novel member of the family Pasteurellaceae, *Emayella augustorita*, was introduced in the August 2024 issue of Emerging Infectious Diseases, by Meyer and colleagues of Limoges, France. Isolated from a patient in France, the bacterium is a new member of the family Pasteurellaceae, named in honor of Louis Pasteur (Figure). Pasteur was deemed by medical historian Robert P. Gaynes to be the “most notable nonphysician in the history of medicine.” His name is most recognized for the eponymous pasteurization process, but he is also lauded in Linnaean taxonomy with the family name Pasteurellaceae, formally accepted in 1981. The designation was conceived to accommodate a collection of gram-negative organisms currently representing 34 genera and 105 species, described as “specialized commensals, primarily and potential pathogens of vertebrates–mainly mammals and birds.”

**Figure:**
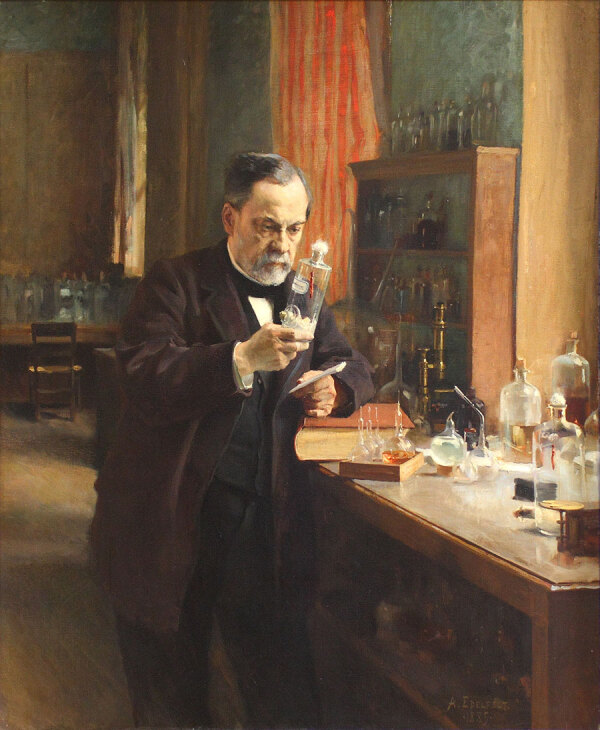
This 1885 painting by Albert Edelfelt (1854–1905), currently in the collection of the Musée d'Orsay (Paris, France), shows Louis Pasteur in his laboratory. Pasteur was an accomplished artist, and examples of his artwork, done as early as age 15, are on display at the Pasteur Institute in Paris. Before pursuing his scientific career, Pasteur aspired to become an art teacher. Image source: Wikimedia Commons.

The constituent bacteria of this family have a propensity to inhabit the mucosal membrane of the mouth, respiratory, and genital tracts. The species *Haemophilus influenzae* and *Aggregatibacter actinomycetemcomitamitans* are well-known Pasteurellaceae that contribute to human illness. Of note, the *Pasteurellaceae* family is polyphyletic, and the taxonomy of some of the species is in need of further study.

## References

[1] Meyer S, Tilloy V, Durand-Fontanier S, Lafon T, Garnier F, Martin C, et al. *Emayella augustorita*, new member of Pasteurellaceae, isolated from blood cultures of septic patient. Emerg Infect Dis. 2024;30:1719–21. 10.3201/eid3008.231651 38907366 PMC11286039

[2] Gaynes RP. Louis Pasteur and the germ theory of disease. In: Germ theory: medical pioneers in infectious diseases. Washington: ASM Press; 2011. p. 143–71.

[3] Leibniz Institute DSMZ—German Collection of Microorganisms and Cell Cultures GmbH. List of Prokaryotic names with Standing in Nomenclature. Family *Pasteurellaceae* [cited 2024 May 18]. https://lpsn.dsmz.de/family/pasteurellaceae

[4] Bisgaard M. Taxonomy of the family Pasteurellaceae Pohl 1981. In: Donachie W, Lainson FA, Hodgson JC, editors. Haemophilus, Actinobacillus, and Pasteurella. Boston: Springer; 1995 [cited 2024 May 18].

[5] Christensen H, Bossé J, Angen Ø, Nørskov-Lauritsen N, Bisgaard M. Immunological and molecular techniques used for determination of serotypes in *Pasteurellaceae*. In: Pavia CS, Gurtler V, editors. Methods in microbiology, vol. 47. Immunological methods in microbiology. Cambridge: Academic Press; 2020 [cited 2024 May 18]. https://www.sciencedirect.com/science/article/pii/S0580951720300027

